# Tackling Tuberculosis in Latvia

**DOI:** 10.1371/journal.pmed.0020122

**Published:** 2005-05-31

**Authors:** Melanie Zipperer

## Abstract

After the fall of the Soviet Union, Latvia faced rising unemployment, poverty, and alcoholism, conditions that fueled a surge in TB. Zipperer discusses how Latvian health professionals are tackling the problem.

With a forceful gesture, the delicate blond unlocks the heavy door of a cell in Riga's Central Prison clinic (Figure 1). Her name is Dr. Inga Nagele. She is the head of the clinic's tuberculosis (TB) unit. She opens the door to check on three female prisoners who have just been admitted. The conditions in the cell are bleak. Washing hangs on a line that stretches from one wall to the other. The bars on the small window hardly let any light into the room, and a bucket in the corner serves as a toilet. All the women were infected with TB at the prison.

**Figure:**
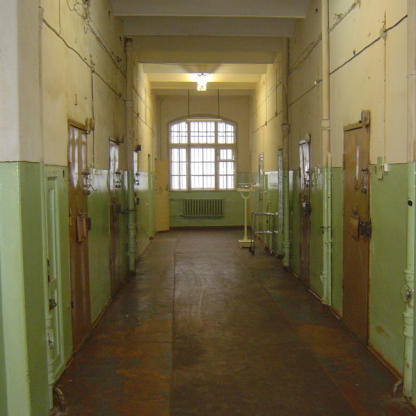
Corridor of the clinic in Riga's Central Prison (Photo: Melanie Zipperer)

“Living conditions in Latvia's prisons are miserable. This is ideal for a relentless spread of the life-threatening lung bacteria and other communicable diseases”, says Dr. Nagele. Because of permanent overcrowding, poor hygiene, and frequent relocation of prisoners within Latvia's 15 prisons, two-thirds of inmates infected with TB developed the disease once they were locked up, she says.

Latvia has 352 prisoners per 100 000 individuals in the population [1]. This number makes it one of the countries with the highest proportion of prisoners in the European Union, according to Mihails Azarenko, Chief of Riga's Central Prison. It is particularly high when compared with Denmark, for example, which has only 64 prisoners per 100 000 individuals [1].

## Rising TB Rate after the Breakdown of the Soviet System

Outside Latvia's prisons, the conditions were just as favourable for an unchecked spread of infectious diseases, and TB in particular. The fall of the Soviet Union in 1991, and with it the collapse of a centralised public-health system, left the population in the newly independent states without proper access to health care. Latvia struggled against soaring unemployment, poverty, and alcoholism. These transition problems led to a rapid increase of TB and the development of multidrug-resistant TB (MDR-TB) strains.

MDR-TB is a form of the disease resistant to at least two of the most powerful conventional antibiotics that can treat TB (rifampicin and isoniazid). It develops when public-health programs fail to deliver regular, reliable treatment to patients. While the common strain of TB can be cured at a cost of US$10 for a six-month treatment course, MDR-TB can cost 100 times as much and takes two years to treat.

According to the World Health Organization's (WHO's) “Global Tuberculosis Control” report [2], reported notifications of TB in Latvia peaked in 1998 with 90 cases per 100 000 individuals. This made Latvia the country with the highest TB incidence rate in the European Union, compared with an average rate of 13 per 100 000 in western Europe. It was also considerably higher than in the other two Baltic states, Estonia (48 per 100 000) and Lithuania (70 per 100 000). Rates of MDR-TB in Latvia are among the highest in the world. According to a report by the WHO and the International Union Against Tuberculosis and Lung Disease, patients in parts of eastern Europe and central Asia are ten times more likely to have MDR-TB than in the rest of the world [3].

Rinalds Mucins, former Health Minister of Latvia, sees the main reason for Latvia's health problems as the difficult transition process of the health system. He was part of a government that resigned on 28 October 2004, when parliament rejected the prime minister's draft budget. “Over the past ten years we have tried every health system from Semashko to Bismarck. We changed our system too many times and too rapidly and couldn't provide patients and doctors with any stability”, he says. “Now we are working on some long-term programs; this is definitely a good start.”

## Implementing DOTS and DOTS-Plus

This good start has already yielded positive results. Since 1995, Latvia has begun to make great strides in reducing the threat of TB and has started to rigorously implement DOTS, the internationally recommended strategy for controlling TB (see Sidebar).

The government backed this up with a strong political and financial commitment, resulting in TB cure rates rising from 64% in 1997 to 73% in 2002, according to the WHO European Regional Office centralised information system for infectious diseases. As a response to the serious MDR-TB situation, the DOTS-Plus project was introduced a couple of years later in close collaboration with the WHO and the United States Centers for Disease Control and Prevention. The DOTS-Plus project was also approved by the Green Light Committee. This committee is the technical review panel of the Green Light Committee mechanism, the WHO's mechanism to enable access to quality-assured second-line drugs at a reduced cost and technical assistance in the management of MDR-TB [4].

DOTS-Plus targets MDR-TB and builds on the elements of the DOTS strategy. However, it also takes into account specific issues, such as the use of second-line drugs, that need to be addressed in areas with a significant prevalence of MDR-TB. “DOTS-Plus has been very effective so far. According to the State Centre of Tuberculosis and Lung Diseases the number of MDR-TB cases decreased by 50%, from 335 cases in 1997 to 166 in 2003”, says Dr. Aiga Rurane, head of the WHO Liaison Office in Riga, “However, much more remains to be done to fully control the spread of MDR-TB.”

In 1997, TB control in prisons became part of Latvia's National TB Control Program. Since then, all prisoners undergo regular examinations and sputum tests. With an 85.9% cure rate, the number of patients with TB who are cured in prisons is now higher than outside. According to official data provided by the State Centre of Tuberculosis and Lung Diseases (http://www.tuberculosis.lv), the number of cases of MDR-TB in prisons was reduced from 19 in 2000 to nine in 2003 [5]. “The reason for these good cure rates is that patients are under permanent surveillance and must swallow the prescribed drugs,” says Dr. Nagele. “It also helps that they get a warm meal and regular sleeping hours.”

Dr. Gunta Dravniece, head of the MDR-TB ward in Riga's State Agency for TB and Lung Diseases, believes that controlling TB inside prisons is key to preventing the spread of the disease in the rest of the country. The State Agency for TB and Lung Diseases is located in a forest, a half-hour drive outside Riga. It was built in the 1950s by prisoners and designed as an isolation hospital for Riga's patients with TB. It has separate sections for patients with TB and MDR-TB.

At least one-third of Dr. Dravniece's patients in the MDR-TB ward are ex-prisoners. The others are often socially disadvantaged, homeless, and suffering from chronic alcoholism. “Many of them don't have even a passport,” she says. “When they get infected with MDR-TB, they want to stay as long as possible here at the state agency clinic, because they have a bed and something to eat.”

Long-term treatment, which is a necessity for MDR-TB, is difficult for both patients and health workers. Patients have to take up to 25 pills every day. Nurse Valentina carefully watches that each of the 55 patients with MDR-TB in the ward takes them. “They don't like these drugs, because often the pills provoke sickness and are difficult to digest. So I have to explain to them over and over why it is so important to take the medication regularly”, she explains.

Once patients are not infectious anymore, they can leave the ward and go back to their hometowns. There they receive almost daily follow-up treatment by local doctors and nurses. “Patients who come for their medical checks get food and are reimbursed for transportation. This is cheaper for the public sector than the hospital cost and motivates them to come back”, says Dr. Dravniece.

## Training Centre to Disseminate Lessons Learned

The state agency also includes a training centre. It offers research and training courses to TB experts from eastern Europe and central Asia. Since 2000, the training centre has held countless courses for program managers, social workers, nurses, and laboratory staff across Europe. Training modules focus on key issues such as improvement of laboratory diagnostic methods, patient education, and MDR-TB in prisons. So far 156 specialists have been trained.

In November 2004, the training centre was officially awarded the status of a WHO Collaborating Centre, the first one on MDR-TB in the European region. “We accumulated a lot of experience in the past years while implementing DOTS and DOTS-Plus”, says Dr. Vaira Leimane, director of the training centre. “We received a lot of help from donors. Now it is time for us to give back. We want to show other countries with similar problems what we learned, so that they can improve their capacity to effectively treat and control the disease.”

## Co-Infection with TB and HIV

However, the battle is not won yet. A new public-health challenge is posed by the increasing number of patients with HIV/TB co-infection. Eastern Europe and central Asia are home to some of the fastest-growing epidemics, mostly among young people. People who are HIV-positive are more likely to become sick with TB and MDR-TB than others. In Latvia, according to the State Centre of TB and Lung Diseases, the number of patients co-infected with HIV and TB has risen from one in 1994 to 40 in 2003.

Worldwide, in 2000, 11% of all new cases of TB in adults occurred in people infected with HIV, and 9% of the cases were directly attributable to HIV. The contribution of TB to AIDS-related deaths is substantial: 11% of AIDS-related deaths occur primarily from TB [5].

Dr. Jack C. Chow, the WHO's Assistant Director-General for HIV/ AIDS, TB, and Malaria, stresses that TB and HIV control programs must collaborate: “There are many ways how TB and HIV programs can closely work together. HIV-positive people can be given prophylactic treatment to prevent development of active TB disease. TB patients can be offered an HIV test.”

Without prompt action in this direction, years of Latvia's successful TB control efforts could be undermined.

**DOTS and DOTS-Plus**

DOTS (for “directly observed treatment, short course”) is the internationally recommended TB control strategy that combines five elements: sustained political commitment; access to quality-assured TB sputum microscopy; standardized short-course chemotherapy to all cases of TB under proper case-management conditions; uninterrupted supply of quality-assured drugs; and a recording and reporting system enabling outcome assessment.

DOTS-Plus takes into account specific additional issues, such as the use of second-line drugs, that need to be addressed in areas where there is high prevalence of MDR-TB. DOTS-Plus works as a supplement to the standard DOTS strategy. By definition, it is impossible to conduct DOTS-Plus in an area without having an effective DOTS-based TB control program in place.

## Abbreviations

MDR-TB: multidrug-resistant tuberculosis
TB: tuberculosis
WHO: World Health Organization
